# Building continuity in handovers with shorter residency duty hours

**DOI:** 10.1186/1472-6920-14-S1-S16

**Published:** 2014-12-11

**Authors:** Vineet M Arora, Darcy A Reed, Kathlyn E Fletcher

**Affiliations:** 1 Department of Medicine, University of Chicago, Pritzker School of Medicine, Chicago, Illinois, USA; 2 Department of Medicine, Mayo Clinic College of Medicine, Rochester, Minnesota, USA; 3 Department of Medicine, Milwaukee VAMC/Medical College of Wisconsin, Milwaukee, Wisconsin, USA

## Abstract

As junior doctors work shorter hours in light of concerns about the harmful effects of fatigue on physician performance and health, it is imperative to consider how to ensure that patient safety is not compromised by breaks in the continuity of care. By reconceptualizing handover as a necessary bridge to continuity, and hence to safer patient care, the model of continuity-enhanced handovers has the potential to allay fears and improve patient care in an era of increasing fragmentation. “Continuity-enhanced handovers” differ from traditional handovers in several key aspects, including quality of information transferred, greater professional responsibility of senders and receivers, and a different philosophy of “coverage.” Continuity during handovers is often achieved through scheduling and staffing to maximize the provision of care by members of the primary team who have first-hand knowledge of patients. In this way, senders and receivers often engage in intra-team handovers, which can result in the accumulation of greater common ground or shared understanding of the patients they collectively care for through a series of repeated interactions. However, because maximizing team continuity is not always possible, other strategies such as cultivating high-performance teams, making handovers active learning opportunities, and monitoring performance during handovers are also important. Medical educators and clinicians should work toward adopting and testing principles of continuity-enhanced handovers in their local practices and share successes so that innovation and learning may spread easily among institutions and practices.

## Background

The reduction of working hours for junior doctors has attracted worldwide attention in recent years, bringing with it an unprecedented focus on handovers. Traditionally an area in which junior doctors have received little or no formal teaching, the handover has now become a major concern for patient safety advocates and medical educators alike. Not surprisingly, the World Health Organization has declared the prevention of handover-related errors to be one of its top five “Patient Safety Solutions,” thus firmly establishing handovers as a priority on the same plane as hand hygiene.[1] Several countries have also made safe handovers a priority. For example, the Junior Doctors Committee of the British Medical Association issued a white paper on best practices for handovers, recognizing the need to move from “personal continuity” to “system responsibility.”[2] Likewise, the Australian Commission on Safety and Quality in Health Care launched its Clinical Communications program with a specific focus on improving handovers.[3] In the United States, the Institute of Medicine issued a report on resident duty hours in which it recommended that all residents receive training in handover communications.[4] Following suit, the Accreditation Council for Graduate Medical Education recently required all programs in the United States to ensure that residents are “competent in communicating with team members in the hand-over process” and to “monitor effective, structured hand-over processes” to ensure they are safe.[5]

Although educational programs that address handovers are starting to be described and disseminated, it is important to consider why handovers have not received this degree of attention until now. Historically, the term “residents” denoted physicians-in-training who actually resided in the hospital and were thus able to provide care to their patients at any hour of the day or night.[6] In an earlier era, residents were forbidden from marrying, to better enable them to focus all of their attention on patients. As a result, handover communication was not a prominent component of medical training. Furthermore, because one of the highest values in the physician-patient relationship is the principle of continuity, transferring the care of patients between doctors was generally avoided. Until now, the concept of “continuity of care” has been premised largely on a singular relationship between a patient and a doctor. Unfortunately, this nostalgic notion is the lens through which many of our current team structures and handovers are still viewed. It is not surprising, therefore, that one of the greatest fears expressed by program directors is that junior doctors working within contemporary duty-hour limitations will not be able to provide optimal continuity of care.[7] Clearly, the definition of continuity needs to evolve in tandem with the emergence of new team structures and new approaches to staff scheduling.

The challenge in shortening resident hours lies in ensuring continuity despite needed periods of rest for those primarily responsible for patient care, namely junior doctors. Therefore, the handover, during which primary responsibility for a patient is transferred from one junior doctor to another, must become—as the notion of transfer implies—the bridge to continuity.[8] Moreover, in this new era, an approach to continuity based on the singular doctor–patient relationship is not feasible for junior doctors, who must leave the hospital to comply with duty-hour restrictions. A new approach that encompasses the entire care team is needed.

**Figure 1:**
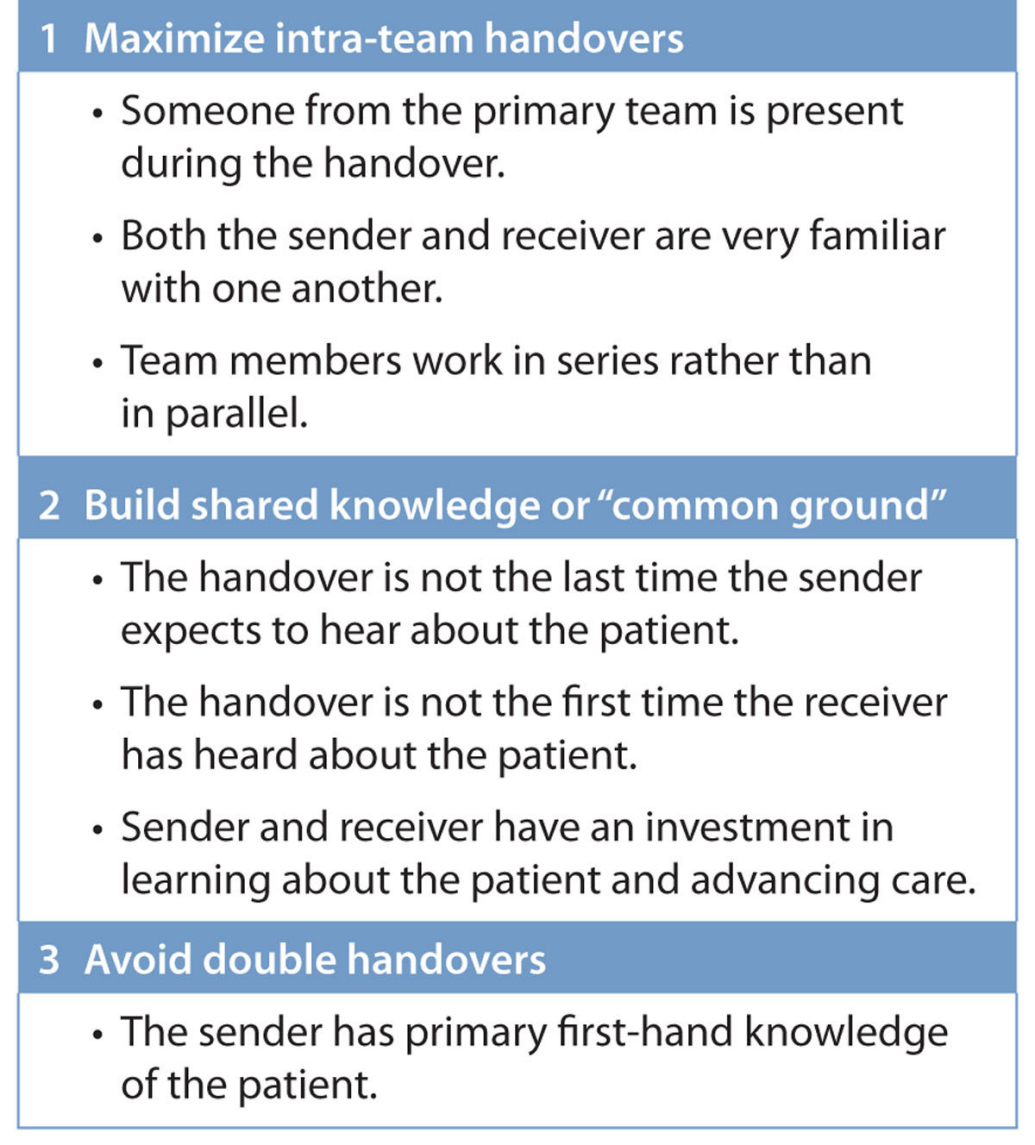
Principles of continuity-enhanced handovers

As contradictory as this may seem, building *system* continuity into handovers—that is, designing “continuity-enhanced handovers”—is one way of ensuring that handovers serve as a reliable bridge to continuity and patient safety. In doing so, we must consider several important principles that maximize team continuity (Fig. 1). That being said, because maximizing team continuity is not always possible, several other strategies that can help both senders and receivers meet their professional responsibilities during handovers are discussed in the following sections.

Table 1 summarizes the changes necessary to convert traditional handovers to continuity-enhanced handovers that provide the necessary bridge to help ensure patient safety in a teaching hospital care setting.

**Table 1:** Traditional versus continuity-enhanced handovers in teaching hospitals

Features	Traditional	Continuity-enhanced
**Transfer of information**	As little information as possible is given, so as not to “burden” cross-cover with any tasks *The primary team sender apologizes to the night float covering physician during handoff: “I got a new patient today and I tried to tuck her in so you should not have any trouble. The only thing is that GI may call with recs.”*	A robust interactive exchange occurs to promote a shared mental model with active conversation *The primary team sender engages the night-float covering physician by sharing her thought process and rationale: “I got a new patient today… she is a 25 yo female who recently completed a course of ciprofloxacin and now has abdominal pain. I ordered a GI consult because of her elevated liver enzymes. I am wondering if she has drug-induced liver injury.”*

**Professional responsibility**	The individual physician or “primary team” transfers responsibility to a “covering physician” *Later that evening the GI consult team calls and asks the night float covering physician whether the patient has had a PT/INR test. The covering physician replies, “This is not my patient.”*	A team of clinicians who share responsibility equally for the patient *Later that evening, the GI consult team calls and asks the night-float covering physician whether the patient has had a PT/INR test. The covering physician says, “I did not hear that during handover, but I will check and get back to you.”*

**Philosophy of “coverage”**	The covering physician temporizes until the primary physician team returns *The next morning, the night float physician says to the primary team, “The consult team called and wanted you to order an abdominal ultrasound.”*	All team members advance care through handover *The next morning, the night float physician says to the primary team, “The consult team called and wanted to order an abdominal ultrasound so I arranged for it this morning.”*

**Learning**	Learning is limited to the individual physician or team dealing with the patient *The primary physician reads about the utility of abdominal ultrasound to diagnose drug-induced liver injury.*	Handovers are used as a learning opportunity for all clinicians present *During handover,the sender and receiver discuss indications for abdominal ultrasound in this patient.*

**Scheduling / staffing**	The time when an individual physician responsible for patient is present is maximized *The primary team sender stays late to meet the family because the covering physician does not know the patient’s situation well enough to meet the family and discuss the patient’s prognosis.*	The time when *any* member of primary care team is present is maximized *The primary team sender can leave the hospital after her shift and hands the patient’s care over to another physician, who is also member of the primary team and meets with family to discuss the patient’s prognosis.*

## Maximizing team continuity

First, *team* continuity must be prioritized over *personal* continuity between individual physicians and patients. One way to achieve this is to design a schedule that maximizes the amount of time during which someone from the primary team (i.e., the team of physicians ultimately responsible for the patient) is present. Maintaining team continuity may decrease the potentially harmful clinical uncertainty that often results from handovers to individuals who are not part of the primary team.[9] Often this will entail scheduling team members to work in series (team members spread over multiple shifts) rather than in parallel (multiple team members on a dedicated shift who then hand off to a “covering physician”). Having team members work in series provides a foundation for an “intra-team” handover to occur, in which the sender and receiver are both part of the primary team and are therefore familiar with the patient. Unfortunately, scheduling a team to provide continuity in this fashion can be challenging when large teams are needed or when staffing levels need to be matched to a fluctuating workload. When it is not possible to maximize continuity across an entire day, it is worth considering how to maximize continuity through “high acuity” times such as the daytime, when patients are seen for tests or procedures and the bulk of clinician communication to advance care occurs.

Second, continuity-enhanced handovers should aim to build some shared knowledge or “common ground” on which an active conversation or dialogue can be based.[10] The more common ground, or shared information, that the sender and receiver have coming into the handover, the more likely it is that an active dialogue will occur. Active conversation is important, as it provide opportunities for assumptions to be questioned and for errors in ongoing management to be detected.[11] Common ground is more easily achieved when the sender and receiver have an opportunity for repeated interaction through the course of a patient’s stay. Ensuring repeated interaction also minimizes the number of handovers in which a sender and receiver exchange information in an isolated fashion (Fig. 1). This way, the receiver will have already made an investment in learning about the patients he or she is receiving. This may also enable senders and receivers to focus on new patients, for whom there has been no time for common ground to be established, since this will be the first time the receiver has heard about the patient.

When intra-team handovers are not possible, it is still important to maximize common ground by avoiding the use of “temporary coverage,” i.e., physicians or other professionals who provide care for a short period before a second handover occurs. Although multiple handovers involving the same patient and providers can also pose a risk, the “double handover,” in which neither the receiver nor the sender have primary knowledge of the patient, is particularly problematic. In lieu of primary knowledge, the sender in this type of handover resorts to reciting the information that he or she recalls from the handover received from the initial sender, who had primary knowledge of the patient. Given the fact that receivers are often unable to recall the most important items that primary senders have communicated, double handovers are especially risky because they make it even more likely for important information to be lost.[12] The double handover may also compromise quality of care if the personal investment of the sender is diminished by the lack of a personal connection or allegiance to the patient. Stricter resident duty hours have given rise to more double handovers, but in view of the unique risks that they present they should be avoided when an alternative is possible. For example, one potential way to reduce double handovers is to stagger start and stop times of members of the same primary team so that a team member who has first-hand knowledge of the patient can stay late to “send” the patient to the night receiver.

Because double handovers do exist, it is important to highlight that written handover documents can help to provide common ground between care providers in certain scenarios. Through the written synopsis of the clinical situation for a patient from the primary team, a receiver can start to formulate a mental model of the patient. This means that having an updated, easy to understand written handover tool is of crucial importance in ensuring patient safety.

## Creating high-performance teams

Although maximizing team continuity is one mechanism to create continuity-enhanced handovers, this can be practically challenging to implement. Moreover, ensuring that members of a team are present does not necessarily mean that they share a common vision or goal regarding their role or responsibility toward the patient’s care. This is in sharp contrast to the concept of high performance teams, in which members espouse a shared vision and common goal. Team members have clear roles, but also preserve an element of flexibility or support such that they can “back each other up” if one member is unexpectedly challenged.[13] High performance teams also engage in performance monitoring.

How would high performance teams handle handovers? The most critical element is for each member to accept professional responsibility for a patient. Phrases such as “This is not my patient” or “I’m just covering” send a clear signal that a physician does not espouse the same sense of professional responsibility demanded by all high-performance team members. These sentiments are also a symptom of the “shift-work” mentality that medical educators loathe.[14] It is especially critical to instill a heightened sense of responsibility among team members whose roles have been added to the patient care team to ensure duty hour compliance. These new team members include the “float” resident (whether day or night), moonlighters, hospitalists, and non-medical professionals. Creating increased accountability among these care providers for the quality and outcomes of care is one way to achieve a shared vision. Given their pivotal role as team leaders, it is essential that teaching attendings articulate the framework of this more broadly defined team and act as effective role models within it. Engaging float residents in this type of vision can be challenging. One strategy that can help is to use models in which team members have clear roles and responsibilities and a strict process is followed. One example is the adaptation of team principles from Formula 1 Racing to the handover.[15] Even though float residents may be meeting patients for the first time, they can still have a clear awareness and understanding of their critical role so they can function as effective team members. Such a shared vision may also be cultivated through briefings to plan the handover, and debriefings about how particular handovers went in order to better understand how handovers should be conducted.

## Making handovers an active learning opportunity

Continuity-enhanced handovers can also provide a conduit for learning. Handovers are associated with clinical uncertainty, making it important for senders and receivers to approach handovers as a way to ameliorate uncertainty through learning.[16] Senders and receivers can engage in an active dialogue to ensure learning of a common approach to specific conditions and diagnoses. Learning may also occur through feedback on clinical actions taken in the preceding shift. This learning is most likely to occur in the setting of high team continuity. For example, if a night-shift physician-in-training admitted a patient with a presumed diagnosis that was confirmed after the shift, he or she would be able to obtain this valuable feedback, thus affirming his or her diagnostic approach to that clinical scenario. This critical feedback could encourage trainees to reflect on the outcomes of clinical actions even when they are not present to see them unfold. This process builds the foundation for expertise based on clinical experience.

In addition to using the clinical communications of patient care as a way to promote learning, there are more formal and direct ways to ensure that learning takes place during handovers. The most obvious of these is the use of senior peers (senior residents) to directly supervise the handover and provide guidance based on their own experience. Although it is also important to recognize that experienced clinicians (faculty, chief residents, etc.) can also supervise the handover, faculty development in this area is likely warranted, since many faculty may have limited experience with new handover processes. The growth of the hospitalist specialism in the United States has also led to handovers becoming a core competency for this group and highlights the need for continued education for practising physicians. [17]

In some fields, attending supervision of “evening rounds” serves as way to ensure not only that communication of critical patient information is occurring, but also that all learners have access to clinical teaching in the context of seeing the patients who are being transferred. When actually seeing patients is not possible, attending supervision can still provide an opportunity to answer the clinical questions that come up during the course of the day. In addition to direct supervision, another possibility is to “formalize” the learning during handovers with short educational modules tailored to a specific case or a common set of cases that are encountered during a service. In doing so, the handover is linked to practice-based learning and improvement, allowing learners to integrate new knowledge into their practice.

## Monitoring performance during handovers

As handovers become more routine and occur at standard start-of-shift and end-of-shift times, routine monitoring of handovers is easier to incorporate as a technique to ensure integrity of the communication. Monitoring handover performance has the added benefit of ensuring that a minimum accepted standard is followed even when intra-team handovers are not possible. Although tools to facilitate performance monitoring are just evolving, some general principles regarding the timing and content of evaluation can be used to guide programs to institute a monitoring plan.

Evaluations can occur in real time, during the handover, or through recall at the end of a rotation. Although this can be challenging to achieve at times, handover is an opportunity to directly observe behaviours related to communication between senders and receivers. In addition, other competencies such as professionalism can be observed. An alternative that can supplement the direct observation of handovers is the use of end-of-rotation evaluations. Although they are subject to recall bias, many junior doctors and faculty physicians are accustomed to retrospective assessments, which also have the advantage of enabling senders and receivers to judge handover performance after repeated interactions, giving them more information on which to base an evaluation.

Handover evaluation should, ideally, be competency-based and linked to specific, observable behaviours. To evaluate the handover sender, the quality of the content delivered can be assessed. For example, was anticipatory guidance included and easy to interpret? Did “to-do” items include a rationale? Evaluating receivers may be more difficult, but observable behaviours could include passive actions such as eye contact or body language. Moreover, active listening, such as with the use of “read-back” or note-taking, is associated with better retention of information.[18] Evaluations can be completed by qualified supervisors, such as chief residents or attending physicians, or by peers who are participating in the handover. Peer evaluation has the added benefit of enabling junior doctors to actively offer input on how to improve the quality of communication during handover to their colleagues.

In addition to monitoring the quality of verbal dialogue between senders and receivers, written documentation to facilitate the handover—often referred to as the “signout”—can be audited for accuracy and readability. Typically, a structured template, sometimes in electronic format, is used to facilitate the transfer of written information during handovers. However, documents that support handovers, whether on paper or electronically generated, are known to have errors, which most often arise from a failure to update the signout. Therefore, examining accuracy of the information in the structured template, and including key elements such as code status or primary care physician, can help to ensure that handover documents are as current as possible.

## Conclusion

As junior doctors work shorter hours in light of concerns about the harmful effects of fatigue on physician performance and health, it is imperative to consider how to ensure that patient safety is not compromised by breaks in the continuity of care. By reconceptualizing handover as a necessary bridge to continuity, and hence to safer patient care, the model of continuity-enhanced handovers has the potential to allay fears and improve patient care in an era of increasing fragmentation. Medical educators and clinicians should work toward adopting and testing principles of continuity-enhanced handovers in their local practices and share successes so that innovation and learning may spread easily among institutions and practices.

## Authors’ contributions

All authors contributed equally to the preparation of this article.

## Competing interests

The authors have received funding from the Accreditation Council of Graduate Medical Education for work related to residency duty hours. Dr. Arora and Dr. Reed have received research grants from the ABIM Foundation. Dr. Arora has also received grant funding from the Agency for Healthcare Research & Quality and the National Institutes on Aging and has received honoraria from numerous hospitals, the Society of Hospital Medicine, and AHRQ Web M&M. Dr. Fletcher has received funding from the VA HSRD and QUERI as well as the National Cancer Institute and the National Institutes on Aging.

## Declarations

Resources and secretariat support for this project was provided by the Royal College. The authors received a grant from the Accreditation Council for Graduate Medical Education to review the literature on residency duty hours. Dr. Arora has received funding from the Agency for Healthcare Research and Quality (AHRQ grant 1R03HS018278-01, Development and Evaluation of a Tool to Evaluate Hand-Off Quality).

This article has been published as part of *BMC Medical Education* Volume 14 Supplement 1, 2014: Resident duty hours across borders: an international perspective. The full contents of the supplement are available online at http://www.biomedcentral.com/bmcmededuc/supplements/14/S1. This article was submitted in 2011 and peer reviewed in 2012. Final acceptance for publication as part of this supplement was in 2014.

Publication of this supplement was supported and funded by the Royal College of Physicians and Surgeons of Canada. The funding agency played no role in the design, in the collection, analysis and interpretation of data; in the writing of the manuscripts; and in the decision to submit the manuscript for publication. The articles have been through the journal's standard peer review process for supplements. The Supplement Editors declare that they have no competing interests.
